# Using lake sediments to reconstruct the indigenous occupation of the Kukulek archeological site and the impact of recent climate change on freshwater ecosystems at St. Lawrence Island, Alaska

**DOI:** 10.1177/09596836261477277

**Published:** 2026-08-24

**Authors:** Neal Michelutti, Katherine Griffiths, Jules M. Blais, Linda E. Kimpe, Janae Rampone, Joshua Kurek, John P. Smol

**Affiliations:** 1 Queen’s University, Kingston, ON, Canada; 2 University of Ottawa, ON, Canada; 3 Mount Allison University, Sackville, NB, Canada

**Keywords:** paleolimnology, archeology, diatoms, chironomids, stable nitrogen isotopes, Arctic ponds

## Abstract

Changing weather patterns during the late-1870s impaired winter hunting success and devastated the historical village of Kukulek at St. Lawrence Island (Alaska), resulting in famine and abandonment of the site. Here, we use dated sediment cores from two freshwater ponds within the catchment of the Kukulek archeological site to track the history of occupation including the 1878–1880 famine, with comparisons to two reference ponds not affected by direct human activity. The study pond nearest the archeological site recorded the highest values of stable nitrogen isotopes (δ^15^N) as well as eutrophic diatom assemblages, which reflect fertilization from nutrients released from marine mammals butchered onshore. The onset of the eutrophication signal may be related to catchment disturbances during the abandonment of the village in 1880, and from the archeological excavation in the mid-1930s. These physical disturbances occurred during a warming period when thawing permafrost and the release of nutrients from previously frozen meat caches and marine mammal remains may have augmented pond fertilization. In the reference ponds, late 19th century climate warming appears to be the primary driver of limnological change with chironomids shifting to warmer-adapted assemblages and diatom taxa reflecting increased habitat complexity that is often documented with longer growing seasons. The information from the reference ponds underscores the importance of considering climate when tracking cultural eutrophication in the Arctic and elsewhere.

## Introduction

Recent climate change in the Western Arctic and Alaska has already impacted the physical environment including permafrost thaw (Osterkamp and Romanovsky, 1999), reductions in summer sea ice (Holland et al., 2006), and coastal erosion (Gorokhovich et al., 2014). Further warming is projected to exacerbate such changes (Barnhart et al., 2014; Brown et al., 2003). Western Alaska experiences considerable variability in regional climate due to the confluence of several semi-decadal to decadal scale climatic oscillations including the Pacific Decadal Oscillation (PDO), El Niño/La Niña events, and the Arctic Oscillation. In the Bering Sea, a change in the PDO in 1976–1977 CE resulted in marked increases in coastal air temperatures (Bieniek et al., 2012; NOAA, 2015). This resulted in a dramatic reorganization of the marine ecosystem (Hunt et al., 2002; Mueter and Litzow, 2008) including northward shifts of the southern ranges of mammals such as the gray whale and Pacific walrus (Moore et al., 2007; Laidre et al., 2008) and a northern expansion of fish species such as the Bering Sea pollock (Hunt et al., 2002). Overlying these natural climate oscillations is a trend toward warmer waters with reduced sea ice extent and stability (Holland et al., 2006), which affects both marine animals and the indigenous communities that rely upon them for food (Huntington et al., 2016; Litzow et al., 2014).

St. Lawrence Island (Alaska) is located in the Bering Sea, just south of the Bering Strait (Figure 1), and is a key component of the Bering Sea ecosystem due to the formation of a polynya, a cold pool of perennially open water, on its leeward side. The St. Lawrence Island Polynya, an epicenter of sea ice formation, is hundreds of kilometers wide and is an important feeding ground for marine mammals, especially for seals and walrus, as they can rest on the ice and feed in the open water during the winter (Grebmeier and Cooper, 1995). Additionally, the polynya sets up ocean currents that transport nutrients to the benthos of the Southwestern Bering Sea. However, this cold pool has been increasing in temperature (Grebmeier et al., 2006) and expanding with the thinning and contraction of sea ice toward the poles (Clement et al., 2004; Rothrock et al., 1999).

**Figure 1.**
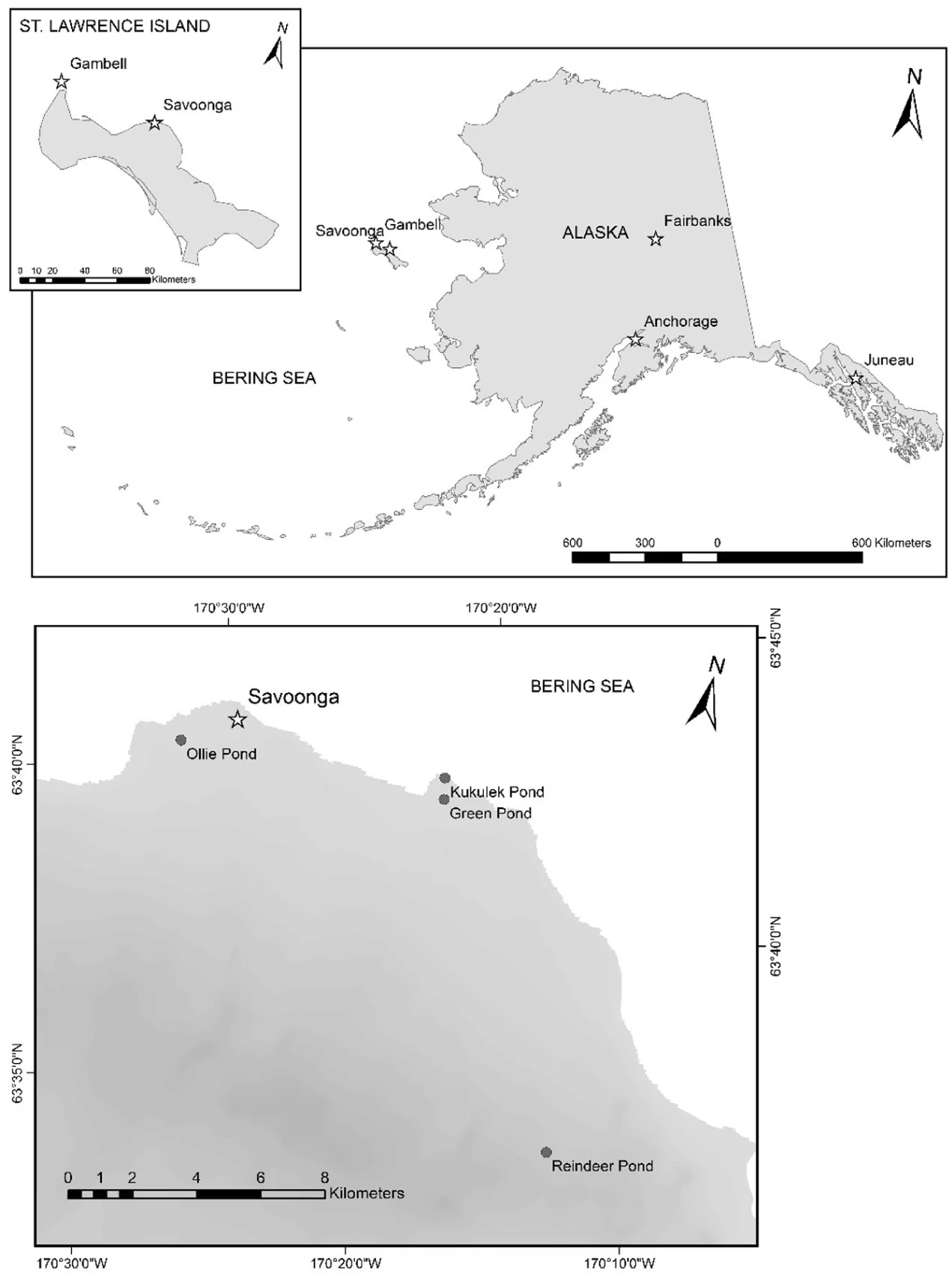
Top: Location map of St. Lawrence Island, Alaska, in relation to mainland Alaska, United States of America. Inset: St. Lawrence Island highlighting our study area (black square) in relation to the towns of Gambell and Savoonga (stars). Bottom: Study region with shaded topographic relief indicating the locations of the four study ponds (dark gray circles).

The native Yupik who live on St. Lawrence Island are predominantly subsistence hunters of marine mammals, primarily of walrus, seals, and whales, and are culturally and economically tied to these resources. This traditional way of life has already been impacted by the changing regional climate (Figure 2) with hunters reporting an increase in warm winter winds and replacement of stable pack and pan ice for brash and thin ice (Grebmeier et al., 2006), which make winter hunts more difficult and unpredictable (Huntington et al., 2016).

**Figure 2.**
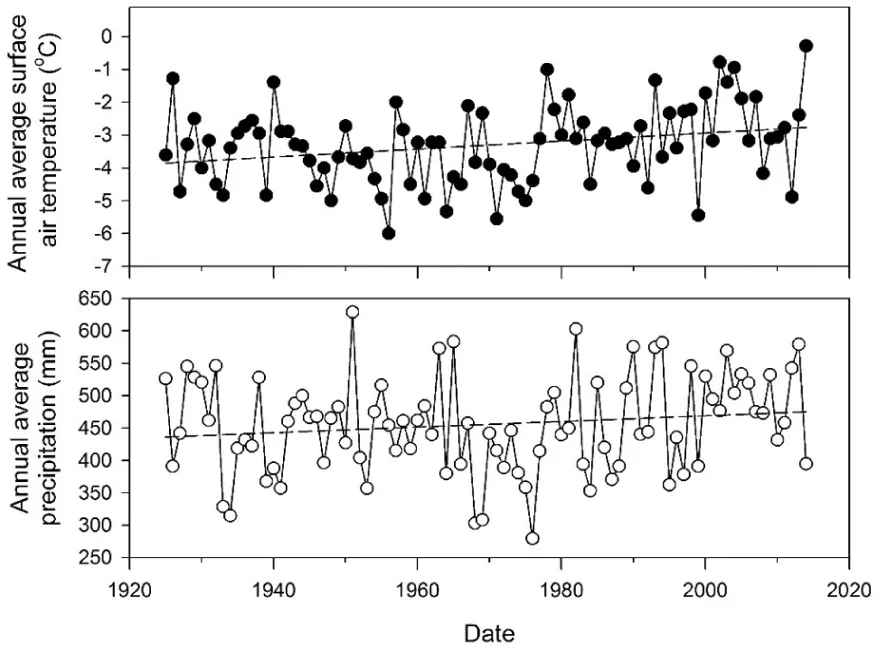
Average annual temperature (top) and precipitation (bottom) records for the western coast of Alaska (climate district 2; Bieniek et al., 2012; NOAA, 2015) including linear regression trend lines (dashed).

The subsistence existence of the Yupik people makes their lifestyle particularly susceptible to climate change. This is perhaps best exemplified by the legacy of Kukulek Village (Figure 1), located on the north–central shore of St. Lawrence Island. The people who occupied the village, like the modern Yupik now, subsisted primarily on a diet of marine mammals, including bowhead and gray whales, various seals, and the Pacific walrus (Geist and Rainey, 1936). In the winter of 1878, a change in weather patterns shifted the dominant winds from the north to predominantly from the south and southeast (Krupnik and Krutak, 2002). The southern winds brought increased storminess, unstable ice, and limited polynya development on the south side of the island, which impaired any hunting by foot or boat during the winter (Krupnik and Krutak, 2002). The lack of access to traditional hunting grounds had a devastating impact on the village, causing famine and, ultimately, abandonment of the site by 1880 (Geist and Rainey, 1936).

Here, we use paleolimnology to track the occupational history at the Kukulek archeological site. Lake sediments are often the only means by which to obtain information on long-term environmental change in remote regions with little-to-no monitoring data. Importantly, lake sediments contain many indicators that track human-environment interactions and have a long history of being incorporated into archeological research (Brown et al., 2022; Patrick, 1938; Tóth et al., 2019; Michelutti et al., 2013). In combination with archeological investigations, paleolimnology can assess the ecological impacts of prior occupations as well as provide a climatic context with which to interpret major human population shifts (Bell and Blais, 2021; Michelutti and Smol, 2019; Viehberg et al., 2021; Wooller et al., 2012, 2018). In Arctic regions, paleolimnological approaches have been used to track earlier cultures primarily via their hunting of marine mammals, which when butchered on land, release nutrients. The release of these marine-derived nutrients results in the fertilization of nearby waterbodies that are typically nutrient-poor ecosystems resulting in trophic cascades and alterations to sediment biogeochemistry (Coleman et al., 2025; Douglas et al., 2004; Hadley et al., 2010a, 2010b).

We analyzed sediment cores from two ponds near the Kukulek archeological site to track the impacts of the historical occupation and abandonment. Specifically, we used stable isotopes of nitrogen (δ^15^N) to track hunting activity because high trophic level marine animals, such as seals and whales, are higher in δ^15^N values relative to terrestrial and freshwater organisms (Duda et al., 2022; Finney et al., 2000). Subfossil diatom algae were used to track alterations to water chemistry caused by fertilization of the ponds because diatoms respond sensitively to nutrients and to habitat changes caused by eutrophication (e.g. proliferation of aquatic mosses; Douglas et al., 2004). Likewise, we tracked changes in whole-lake production via sediment chlorophyll *a* (chl-*a*) concentrations, which increase under the presence of greater nutrients (Michelutti and Smol, 2016). We analyzed subfossil chironomids (non-biting midges), which do not respond directly to nutrients but are often linked to eutrophication-related variables such as low bottom water oxygen and changes to benthic habitat (Brodersen et al., 2004; Brodersen and Quinlan, 2006; Langdon et al., 2006). Chironomid assemblages were also used to track changes in climate as they have species-specific tolerances to water temperature (Eggermont and Heiri, 2012; Porinchu et al., 2009; Walker et al., 1991).

In addition to the two study ponds impacted by the historical occupation, we analyzed sediment cores from two distant reference ponds unaffected by the archeological site to disentangle the effects of cultural eutrophication and climate change. The response of freshwater ponds to climate change has implications to the quality and quantity of freshwater ecosystems on St. Lawrence Island. Overall, this study aims to track the influence of the Kukulek occupation on freshwater ecosystems within the context of changing climatic conditions. Given the rapid pace of climate change in the western Arctic, these data have relevance to the current population on St. Lawrence Island, especially regarding issues surrounding food security and cultural identity.

## Site description

St. Lawrence Island (63.4°N, 170.16°W) is a large (~4600 km^2^), volcanic, Alaskan island located in the Bering Sea, just south of the Bering Strait in the western Arctic (Figure 1). Three mountain ranges dominate the island (an eastern chain; the central, Kookoolgit Range; and the western, Poovoot Range), which are connected by extensive, sandy lowlands (Colinvaux, 1967). All study sites are located on the central range, which is composed of Quaternary alkali-olivine basalts (Patton et al., 2011). Shallow rivers and lagoons are abundant in the lowlands, with some smaller lakes occurring in the rocky, barren outcrops between peaks. The island, once underlain by continuous permafrost with a 60–75 cm deep active layer (Mikulski, 2009) is now underlain by discontinuous permafrost (Olefeldt et al., 2016). Permaforst thawing due to rising temperatures has resulted in nearly ¼ of the island’s lakes experiencing drainage events (Liu et al., 2024). The majority of lake drainage events have occurred in the southern lowland tundra region. There are two main coastal settlements on the island: Gambell, in the northwest, and Savoonga, on the north-central part of the island. The remains of the abandoned village of Kukulek are located on a cape ~7 km to the east of Savoonga that juts out into the north Bering Sea (Figure 1).

Kukulek was occupied until 1880 CE and may have been settled as early as 2000 BCE (Geist and Rainey, 1936). The six main cultural phases of occupation listed from ancient to modern (identified based on harpoon tip structure; Geist and Rainey, 1936) are the Old Bearing Sea, Birnirk, Punuk, Thule, Recent-Prehistoric, and Modern cultures. Archeological excavations of Kukulek are detailed in Geist and Rainey (1936). Briefly, archeological excavations of the two mounds that comprise the Kukulek site commenced in 1934–1935, although initial test cuts were made in 1931, 1932, and 1933. Prior to excavation, the mounds appeared as long hills with the outlines of past modern structures on the surface, overgrown with tundra vegetation. All structures in the eastern mound, west of the test cut, were left intact. On the western mound, 37 meat cache structures, nine houses and one storm shed were uncovered and removed or partially removed where possible. Excavated material was placed on the beach side of the mounds to prevent further erosion of the site.

Two ponds, unofficially called Kukulek Pond and Green Pond, are located just to the south of the abandoned village (Figure 1) and are eutrophic (Table 1) with mossy catchments and abundant aquatic macrophytes.

**Table 1.** Select limnological variables for the four study sites on St. Lawrence Island (Alaska), sampled between 15 and 22 July 2012.

Lake name	Latitude (°N)	Longitude (°W)	Elevation (m a.s.l.)	Depth (m)	pH	Na (mg/L)	Ca (mg/L)	Chl *a* (µg/L)	DOC (mg/L)	DIC (mg/L)	TP_u_ (µg/L)	TN_f_ (mg/L)
Kukulek Pond	63.69116	170.34416	4	0.4	6.7	51.60	2.98	10.5	19.1	2.2	466.0	1.320
Green Pond	63.68529	170.34140	8	0.7	6.0	12.00	0.63	1.2	11.4	0.8	35.9	0.690
Ollie Pond	63.68343	170.51135	15	1.2	7.5	10.00	1.44	3.6	10.6	5.1	54.5	0.829
Reindeer Pond	63.59690	170.22511	320	0.5	7.6	2.75	0.45	<0.1	1.1	1.1	10.8	0.154

Variables of particular interest include sodium (Na), calcium (Ca), water column chlorophyll a (Chl a), dissolved organic carbon (DOC), dissolved inorganic carbon (DIC), total phosphorus–unfiltered (TP_u_) and total nitrogen – filtered (TN_f_).

### Impacted site – Kukulek Pond

Kukulek Pond (unofficial name) is a small (~5 × 5 m), shallow (Z_max_ = 0.4 m) waterbody that drains the historical Kukulek village. It is located within a wetland complex on the south side of the archeological mounds. The wetland is dominated by *Hippurius* cf. *laneceolata*, with some forbs and grasses, and has been periodically drained in recent years to facilitate crab harvests (Savoonga elder, personal communication). It was also drained in 1934 to facilitate the archeological excavation at Kukulek. At the time of core sampling, the pond was hypereutrophic, slightly acidic, and humic (Table 1). Due to its proximity to the ocean (~90 m), storms and sea spray influence the pond resulting in elevated salinity (Table 1).

### Potentially impacted site – Green Pond

Green Pond (unofficial name) is located just south of Kukulek Pond, ~750 m from the old village, and may be hydrologically connected to the wetland at high stands. However, the Kukulek site is not within the catchment of Green Pond, nor does the pond receive direct drainage from the archeological site. Consequently, we have identified Green Pond as a “potentially impacted” site, as it would only receive marine-derived nutrients if or when it was hydrologically connected to Kukulek Pond. Green Pond is located ~300 m from the nearest shoreline and is small (~93 × 36 m) and shallow (Z_max_ = 0.7 m) with a grassy catchment. In the catchment is an abandoned fox trap, and emergent *Hippurius* cf. *laneceolata* and grasses are present at both inlet and outlet. The pond is slightly acidic, humic, and eutrophic (Table 1).

### Reference sites – Ollie and Reindeer ponds

Ollie Pond (unofficial name) is small, round (~50 × 50 m), circumneutral (Table 1), and the deepest of our sampled ponds (Z_max_ = 1.2 m), with a grassy catchment and submerged aquatic mosses and grasses around its border. It has a similar elevation to the study ponds near the archeological site and is located 1.7 km west of Savoonga (Figure 1). Ollie Pond is ~300 m from the end of the Savoonga airport runway, ~800 m from the Savoonga sewage lagoons (est. 1997 CE) and ~500 m from the coast. It is eutrophic and humic, with similar water chemistry to Green Pond (Table 1).

Reindeer Pond (unofficial name) is an elongate (~124 x 360 m) waterbody occupying a higher elevation (320 m a.s.l.) than the other three sites. It is located ~4 km from the coast (Figure 1) in a predominantly rocky, volcanic catchment, with emergent grasses at its inlet and outlet. The northern shore is a pebble and boulder beach about 20 m wide, and a rocky hill lies to the south. A snowbank was present on the southern shore at the time of sampling in mid-July 2015 and the pond’s water level was low as evidenced by an exposed littoral zone of ~10 m width. Tracks left by reindeer, which were introduced to the island in 1900 CE for hunting by the Yupik, were present in the catchment at the time of sampling. The pond water had low dissolved organic carbon, was circumneutral, oligotrophic (Table 1), and had abundant, heavily pigmented *Daphnia* species.

## Methods

### Sample collection and dating

Water chemistry and sediment cores were collected from all study sites during mid-July 2015. Water samples were collected at each site and analyzed for a suite of chemical variables, following the field procedures outlined in Antoniades et al. (2003), at the National Laboratory for Environmental Testing in Burlington, ON, Canada. Sediment cores were collected from the deepest part of the basins using a 3″ (7.62 cm) diameter push corer (Glew et al., 2001). The sediment cores were sectioned onsite into 0.25 cm intervals using a Glew (1988) extruder and kept at 4ºC until they were transported to the Paleoecological Environmental Assessment and Research Laboratory (PEARL) at Queen’s University (Kingston, ON, Canada), where they were frozen and stored. Sediment intervals were freeze-dried in preparation for radioisotopic dating by gamma spectrometry using ^210^Pb (Appleby, 2001) and ^137^Cs activities. Core chronologies were developed using the Constant Rate of Supply (CRS) model in the ScienTissiME package for MatLab^®^ (ScheerSoftwareSolutions, Barry’s Bay, ON, Canada).

### Sediment processing

Sediment intervals were processed using standard techniques for diatoms (Wilson et al., 1996) and chironomids (Walker, 2001). For diatoms, a minimum of 350 valves were counted for each interval and identified to species with reference to Krammer and Lange-Bertalot (1986–1991) and Antoniades et al. (2008). Chironomids were identified to tribe, genus, or species-type, if possible, following Wiederholm (1983) and Brooks et al. (2007). A minimum of 50 whole chironomid head capsules required for adequate ecological assessment (Quinlan and Smol, 2001) were counted for each interval where feasible but counts that did not meet this criterion were marked on the profiles in gray. For both chironomids and diatoms, counts were converted into relative abundances and taxa present at >5% abundance were plotted in relative abundance diagrams, while those occurring at <5% were grouped as “other taxa.”

### Sedimentary chlorophyll a and isotopic analysis

Stable isotopes of nitrogen (δ^15^N) and carbon (δ^13^C), and the percent carbon (%C) and nitrogen (%N), were analyzed at the Ján Veizer Stable Isotope Laboratory (formerly G.G. Hatch SIL Laboratory) at the University of Ottawa (Ottawa, ON, Canada) on freeze-dried bulk sediment using a Micro Cube elemental analyzer. Subsamples were acidified by repeatedly adding 6N HCl to the sediment sample and oven-heated for 20 min until no effervescence was observed for two consecutive acid additions. The added acid increased from 10 to 50 μL and the oven temperature increased from 40°C to 60°C over the course of the repetitions. Acidified samples were analyzed for organic δ^13^C and unacidified samples were analyzed for δ^15^N. Samples were normalized to the following internal standards: C-51 nicotiamide (δ^15^N: 0.07 ‰, δ^13^C: −22.95 ‰), C-52 ammonium sulfate and sucrose (δ^15^N: 16.58 ‰, δ^13^C: −11.94 ‰), and C-54 caffeine (δ^15^N: −16.61 ‰, δ^13^C: −34.46 ‰). The blind standard was C-55 glutamic acid (δ^15^N: −3.98 ‰, δ^13^C: −28.53 ‰). The stable isotope data are reported in standard delta notation (δ); the units are per mil (‰) and defined as δ = ((Rx-Rstd))/Rstd) × 1000 where R is the ratio of the abundance of the heavy to the light isotope, x denotes sample, and std is an abbreviation for standard. δ^15^N values were calibrated to the following international standards: IAEA-N1 (0.4 ‰), IAEA-N2 (20.3 ‰), USGS-40 (−4.52 ‰), and USGS-41 (47.57 ‰). δ^13^C were calibrated to the following international standards: IAEA-CH-6 (−10.4 ‰), NBS-22 (−29.91 ‰), USGS-40 (−26.24 ‰), and USGS-41 (37.76 ‰). Analytical precision was ±0.2 ‰ using glutamic acid.

Visible range spectroscopy-inferred chlorophyll *a* (hereafter VRS chl *a*) was applied down core to track changes in primary production, following methods outlined in Michelutti et al. (2010). Sediments were freeze-dried, then sieved through a 125-µm mesh sieve and analyzed using a FOSS NIRSystem Model 6500 rapid content analyzer. Sedimentary VRS chl *a* concentrations were inferred using log transformed data from Michelutti et al. (2010) with the equation: chl *a* + derivatives = EXP (0.83784 * LN (peak area 650–700 nm) + (−2.48861)).

### Data analysis

Relative abundance profiles for each pond were produced with C2 version 1.7.2 (Juggins, 2007) and major taxa were organized by species scores based on a principal component analysis (PCA) run in the “vegan” package v. 2.0-10 (Oksanen et al., 2013) for the R software environment (R Core Team, 2013). Chironomid and diatom profiles were also summarized using principal component analysis (PCA) on square-root transformed relative abundance data of all taxa. Stratigraphic zones were identified using all taxa with constrained, incremental sum-of-squares cluster (CONISS) analyses and the significance of the zones were determined using a broken stick model within the “vegan” package (Oksanen et al., 2013). Where applicable, breakpoints in the %N, %C, δ^15^N and δ^13^C profiles were determined by applying breakpoint analyses using the “segmented” package (Muggeo, 2008). PCA axis 1 site scores of the chironomid and diatom records for all study ponds (Reindeer Pond, Ollie Pond, Green Pond, and Kukulek Pond), in addition to the VRS chl *a* and δ^15^N, were plotted according to Constant Rate of Supply age-depth models obtained by ^210^Pb gamma spectroscopy using C2 version 1.7.2 (Juggins, 2007) to allow for comparisons across ponds over the past ~300 years.

## Results

### Age-depth models

Given the many reversals in excess ^210^Pb activity (with exception of Reindeer Pond), the CRS model of ^210^Pb deposition was the most applicable and was applied to all study cores (Figure 3). The ^210^Pb activities were high relative to other Arctic sites (e.g. Michelutti et al., 2009; Wolfe et al., 2004), giving us confidence in our age models; however, only in Ollie Pond was the ^137^Cs peak in good agreement with the ^210^Pb dates (corresponding to a CRS date of 1974; Figure 3III). As done in many similar studies, we place more emphasis on the ^210^Pb dates as ^137^Cs is often mobile in organic sediments, such as these.

**Figure 3.**
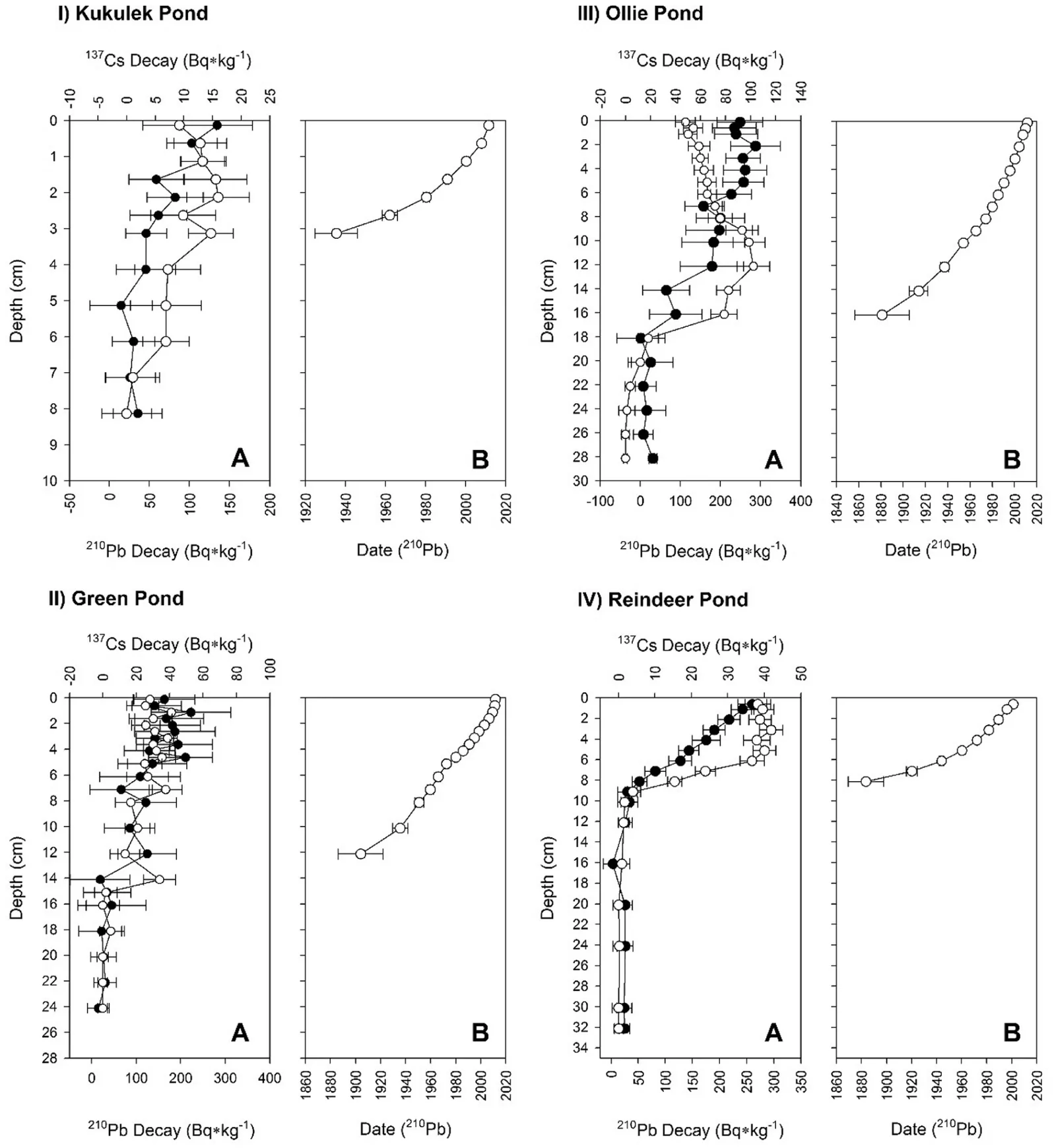
The activity of ^210^Pb (black circles) and ^137^Cs (white circles) versus sediment depth (a) and the Constant Rate of Supply ^210^Pb generated age-depth model (b) for each study site: Kukulek Pond (I), Green Pond (II), Ollie Pond (III) and Reindeer Pond (IV). Error bars represent standard error.

### Kukulek Pond – Impacted site

The subfossil diatoms from Kukulek Pond exhibited an assemblage shift at 5.25 cm (ca. 1787 extrapolated age), from CONISS Zone 1 to Zone 2, and a continuation of this transition from Zone 2 to Zone 3 at 3.25 cm (ca. 1930 CE). Specifically, increases in *Stauroforma exiguiformis*, *Fragilaria capucina*, *Nitzschia homburgensis*, *Nitzschia palea*, *Nitzschia* spp., *Navicula monoculata*, *Achnanthes amoena*, *Diatoma moniliformis*, and *Fistulifera* spp. (Figure 4a) corresponded to declines in *Navicula rhynchocephala*, *Stauroneis* spp., *Diadesmis contenta*, and *Eunotia* spp. The subfossil chironomids showed more muted changes and no significant zones were evident. Around 5.25 cm depth there were minor increases in *Cladotanytarsus mancus*-type, *Corynoneura* spp., and *Psectrocladius* spp. and declines of *Chironomus* spp. and *Metriocnemus eurynotus*-type (Figure 4b).

**Figure 4.**
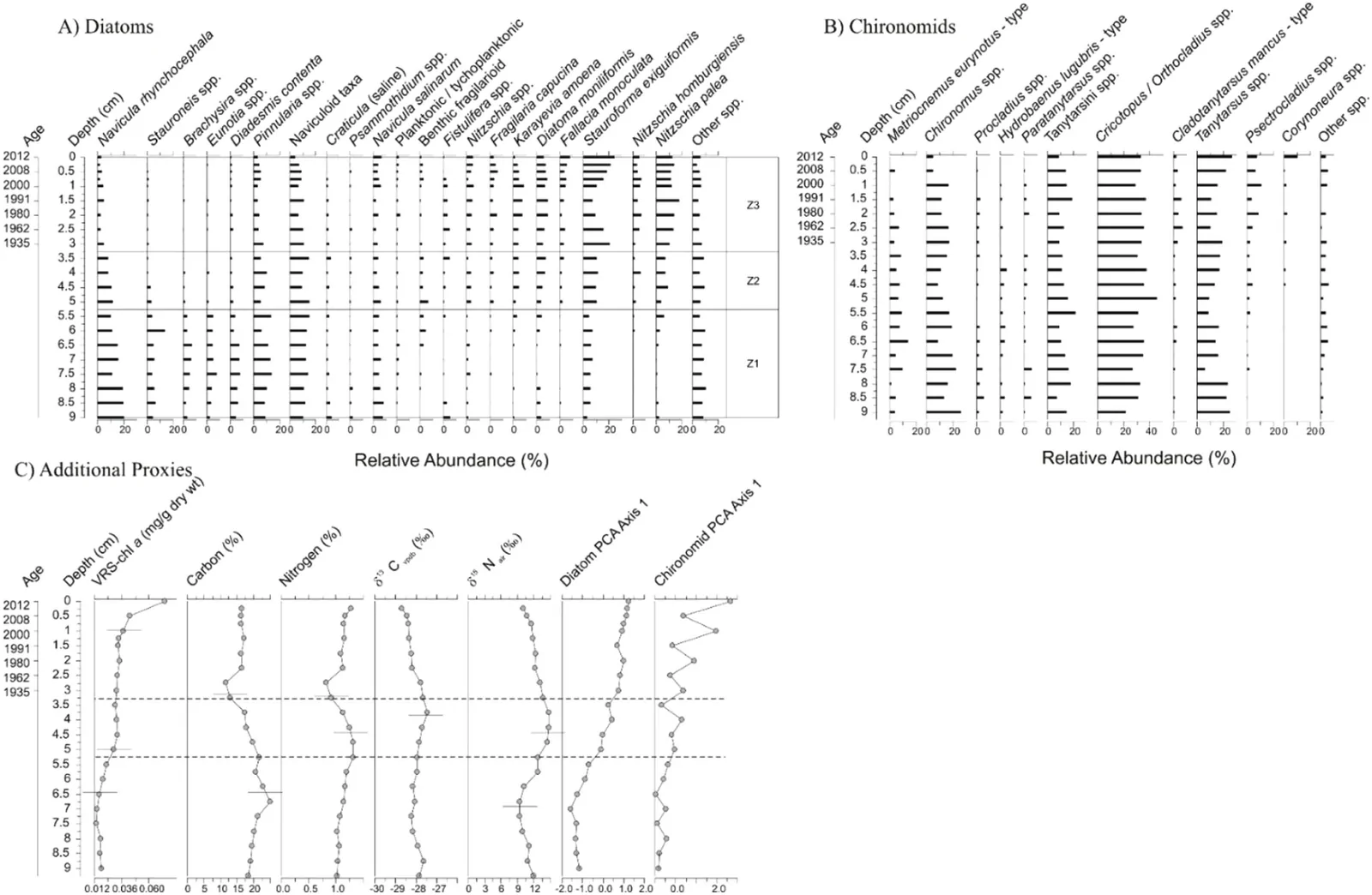
Kukulek Pond, St. Lawrence Island, Alaska, relative frequency profiles of (a) diatom and (b) chironomid taxa sorted according to principal components (PCA) species axis 1 scores. Significant stratigraphic zones, identified using constrained, incremental sum-of-squares cluster analysis (CONISS), are indicated (Z1 – Z3) on the profiles. The profiles of additional proxies (c) include visible range spectroscopy-inferred chlorophyll *a* (VRS chl *a*), percent carbon and percent nitrogen, stable isotopes of carbon (δ^13^C, relative to the standard Vienna Pee Dee Belemnite (V-PDB)) and nitrogen (δ^15^N values, relative to the standard air), with the PCA axis 1 site scores for the diatom and chironomid profiles provided for reference. The significant diatom CONISS zones are indicated by the gray dashed lines while breakpoints are marked on each profile as a short, solid gray line. All profiles (a–c) are plotted against depth and ^210^Pb age.

The sediment chl *a* profile (Figure 4c) showed a minor increase around 5.25 cm depth that gradually increased toward the surface. This is generally coincident with diatom and chironomid species changes as summarized by their PCA axis 1 scores (Figure 4c). Percent carbon and nitrogen correlated poorly with the other proxies, with a period of decline occurring between 6.5 and 3 cm (Figure 4c). Sediment δ^15^N values in Kukulek Pond were highest during diatom Zone 2, reaching a peak of nearly 15‰ and decreasing after 4.5 cm (Figure 4c). The δ^15^N average was 12‰ over the record. The δ^13^C profile was relatively stable until 3.75 cm (one interval before the diatom Zone 2 to Zone 3 transition), after which it declined (Figure 4c).

### Green Pond – Potentially impacted site

The Green Pond diatom stratigraphy (Figure 5a) contains two significant zones. The Zone 1 (28–13.5 cm) to Zone 2 (13.5–0 cm; ca. 1890 CE to present) transition was marked by the declines of *Diadesmis contenta*, *Diadesmis gallica*, *Chamaepinnularia soehrensis*, *Eunotia incisa*, *Boreozonacola hustedii*, and, to a lesser extent, *Brachysira calcicola* and *Nitzschia perminuta*, with corresponding increases in *Psammothidium* spp. (including *P. helveticum*, *P. subatomoides*), *Platessa lutheri*, *Eunotia bilinaris*, *Eunotia praerupta*, *Eunotia silvahercynia*, *Nitzschia palea*, and *Tabellaria flocculosa*. Assemblage shifts in the chironomid record also occurred near 13.5 cm depth, most notably a decline in *Zalutschia* spp. and *Paratanytarsus* spp. and a gradual increase in *Tanytarsus* spp. (Figure 5b).

**Figure 5.**
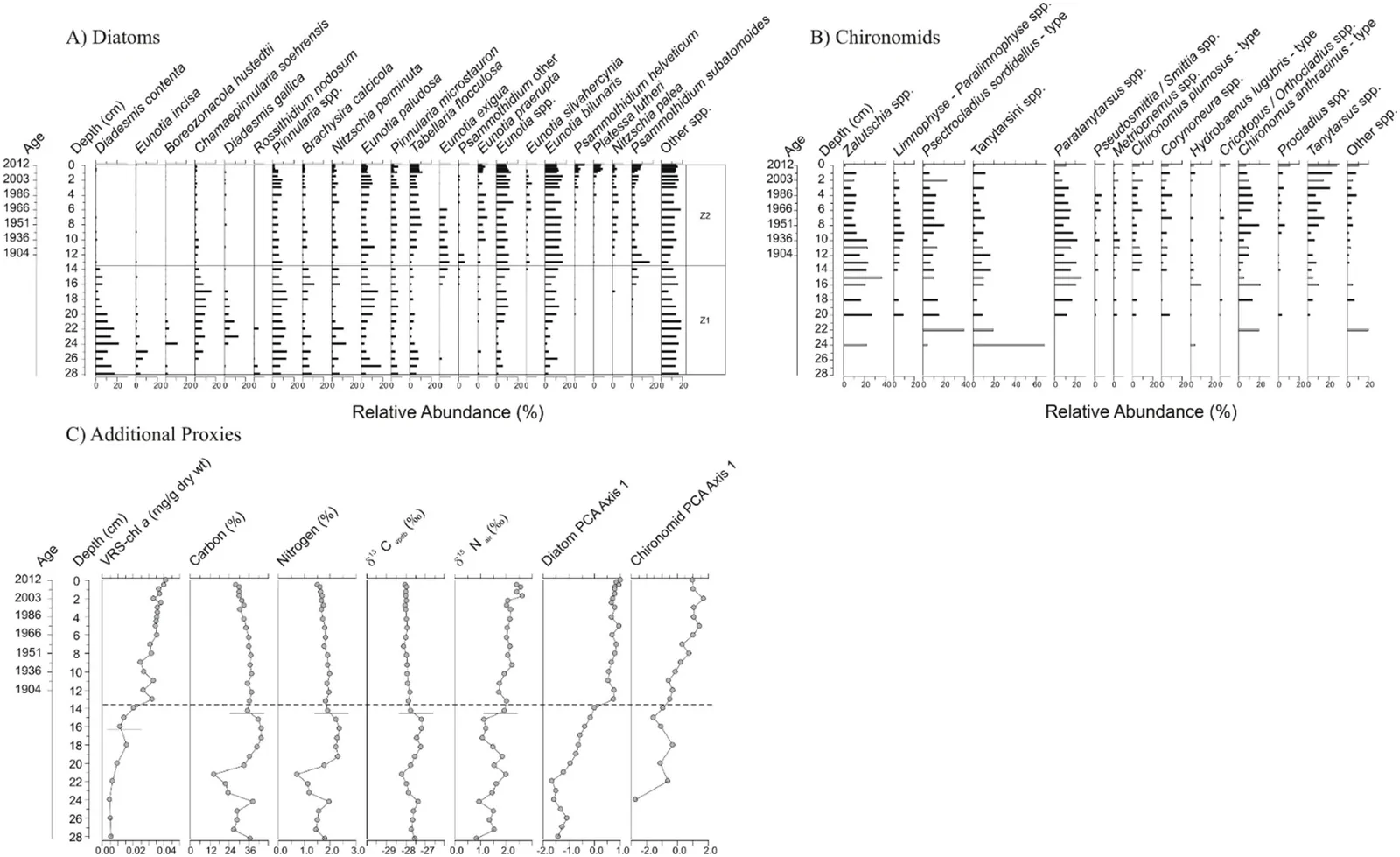
Green Pond, St. Lawrence Island, Alaska, relative frequency profiles of (a) diatom and (b) chironomid taxa sorted according to principal components (PCA) species axis 1 scores. Significant stratigraphic zones, identified using constrained, incremental sum-of-squares cluster analysis (CONISS), are indicated (Z1 – Z2) on the profiles. Gray bars indicate that the interval did not meet the minimum counts. The profile of additional proxies (c) include visible reflectance spectroscopy-inferred chlorophyll *a* (VRS chl *a*), percent carbon and percent nitrogen, stable isotopes of carbon (δ^13^C, relative to the standard Vienna Pee Dee Belemnite (V-PDB)) and nitrogen (δ^15^N values, relative to the standard air), with the PCA axis 1 site scores for the diatom and chironomid profiles provided for reference. The significant diatom CONISS zones are indicated by the gray dashed line while breakpoints are marked on each profile as a short, solid gray line. All profiles (a–c) are plotted against depth and ^210^Pb age.

The VRS chl *a* profile (Figure 5c) gradually increased from the core bottom to 13 cm depth, after which values generally stabilized with a slight increasing trend toward the surface. This is similar to the trend in the diatom PCA axis 1 scores (Figure 5c). Percent carbon and percent nitrogen in the record were variable from the core base to 14.25 cm depth after which the profiles became relatively stable (Figure 5c). Sediment δ^15^N values were notably lower than in Kukulek Pond (mean value 1.8‰) and showed a modest increase toward the surface of the core (Figure 5c). The δ^13^C profile was variable (with changes of ±0.5‰) from the base of the core to 14.25 cm, after which values gradually decreased (Figure 5c).

### Ollie Pond – Lowland reference site

The lowland reference site, Ollie Pond, recorded a relatively stable diatom assemblage over the period of record (Figure 6a). Although there were no significant diatom zones, a slight shift occurred around 16 cm depth (ca. 1880 CE) with modest increases in *Psammothidium rossii*, *Eolimna minima*, and *Achnanthidium minutissimum*. At 6.5 cm depth (ca. 1980s), *Diatoma tenuis* also increased from near negligible abundances to consistently being present, albeit at low relative abundances (<5%). The chironomid profile recorded a significant shift at 13 cm depth (ca. 1920 CE), where the Zone 1 assemblage, dominated by *Zalutschia*-type B declined in abundance toward a more varied assemblage including increases in *Chironomus anthracinus*, *C. plumosus*, and *Procladius* spp. (Figure 6b). We note that at the time of identification, *Zalutschia*-type B refers to the example in Barley et al. (2006), but given that this is a European morphoptype, it is more likely our taxon is *Zalutschia* nr *lingulata pauca* (Medeiros and Quinlan, 2011).

**Figure 6.**
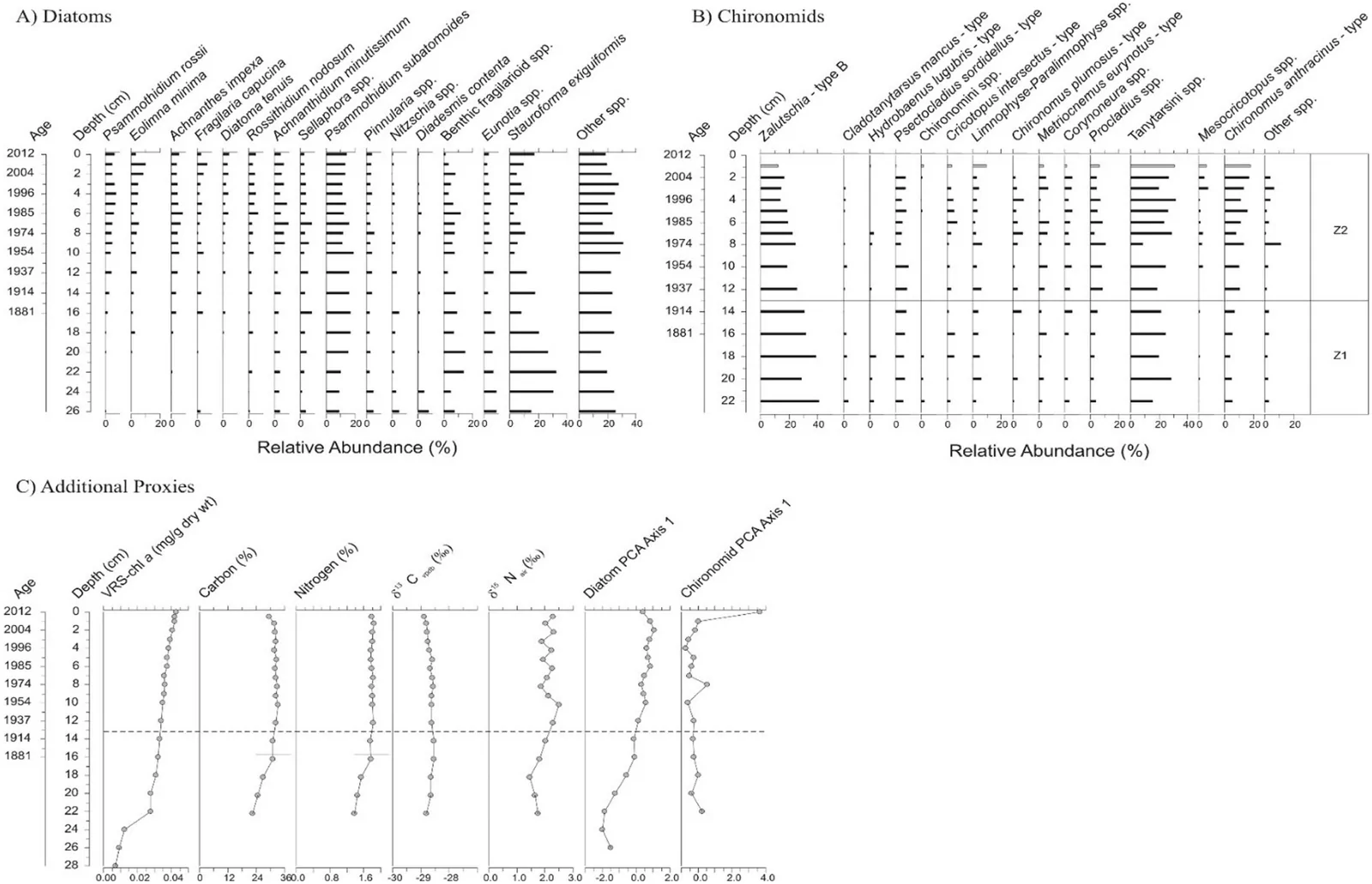
Ollie Pond, St. Lawrence Island, Alaska, relative frequency profiles of (a) diatom and (b) chironomid taxa sorted according to principal components (PCA) species axis 1 scores. Significant stratigraphic zones, identified using constrained, incremental sum-of-squares cluster analysis (CONISS), are indicated (Z1 – Z2) on the profiles. Gray bars indicate that the interval did not meet the minimum counts. The profile of additional proxies (c) include visible reflectance spectroscopy-inferred chlorophyll *a* (VRS chl *a*), percent carbon and percent nitrogen, stable isotopes of carbon (δ^13^C, relative to the standard Vienna Pee Dee Belemnite (V-PDB)) and nitrogen (δ^15^N values, relative to the standard air), with the PCA axis 1 site scores for the diatom and chironomid profiles provided for reference. The significant chironomid CONISS zones are indicated by the gray dashed line while breakpoints are marked on each profile as a short, solid gray line. All profiles (a–c) are plotted against depth and ^210^Pb age.

The chl *a* profile steadily increased over time, as did the percent carbon and nitrogen (Figure 6c). Sedimentary δ^15^N and δ^13^C values were relatively constant throughout the core (mean δ^15^N = 2.03 ± 0.3‰; mean δ^13^C = -28.7 ± 0.1‰; Figure 6c). Sedimentary δ^15^N values were also lower in Ollie Pond than in Kukulek Pond.

### Reindeer Pond – Upland reference site

The diatom assemblages from Reindeer Pond showed modest shifts near the 10 cm depth interval, with declines in the relative abundances of *Stauroforma exiguiformis* and *Fragilaria capucina*, and relative increases in *Achnanthidium minutissimum* (Figure 7a). Reindeer Pond had marked changes in the chironomid record despite no significant zones identified (Figure 7b). This was likely a result of the low counts from 10 to 18 cm depth, which imparted high variability in relative abundances in the lower part of the record. The chironomids identified in the older sediments, including *Arctodiamesa* spp., *Thienemanniella*-type E, *Stempellinella-Zavrelia* spp., *Mesocricotopus* spp., *Parakiefferiella triquetra*-type, and *Hydrobaenus lugubris-*type, were notably different from those in the younger intervals. These taxa, with the exception of *Parakiefferiella triquetra*-type and *Hydrobaenus lugubris*-type, were absent or at low abundances above 10 cm depth, after which chironomid abundances in the sediment became high enough to obtain reliable counts. Around 6.5 cm depth (ca. 1930 CE), there was a decline in *Hydrobaenus lugubris*-type and an increase in *Cricotopus-*type P, *Cricotopus intersectus*-type, *Metriocnemus* spp., *Psectrocladius sordidellus*-type, and Tanytarsini spp.

**Figure 7.**
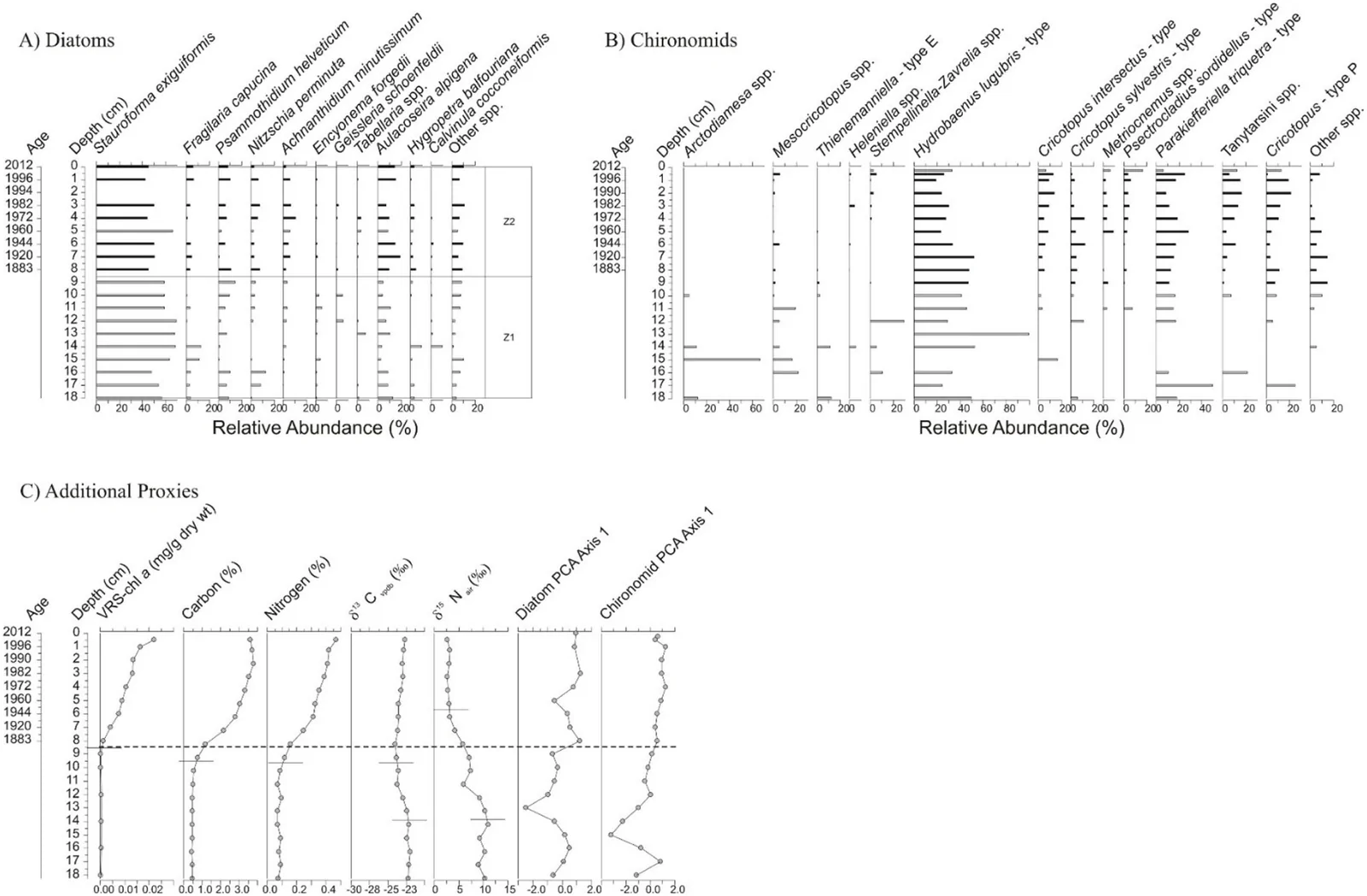
Reindeer Pond, St. Lawrence Island, Alaska, relative frequency profiles of (a) diatom and (b) chironomid taxa sorted according to principal components (PCA) species axis 1 scores. Significant stratigraphic zones, identified using constrained, incremental sum-of-squares cluster analysis (CONISS), are indicated (Z1 – Z2) on the profiles. Gray bars indicate that the interval did not meet the minimum counts. The profile of additional proxies (c) include visible reflectance spectroscopy-inferred chlorophyll a (VRS chl a), percent carbon and percent nitrogen, stable isotopes of carbon (δ^13^C, relative to the standard Vienna Pee Dee Belemnite (V-PDB)) and nitrogen (δ^15^N values, relative to the standard air), with the PCA axis 1 site scores for the diatom and chironomid profiles provided for reference. The significant diatom CONISS zones are indicated by the gray dashed line while breakpoints are marked on each profile as a short, solid gray line. All profiles (a–c) are plotted against depth and ^210^Pb age.

Sediment chl-*a* increased from near zero values below 8.5 cm depth (ca. 1880 CE) to the surface, with percent carbon and nitrogen showing similar trends (Figure 7c). The δ^15^N profile is divided into three segments by two breakpoints. The oldest segment recorded stable values of ~10.0‰ from the base of the core to 13.75 cm, after which values declined from 13.75 to 5.5 cm depth (ca. 1950 CE), and then stabilized around 2.9‰ from 5.5 cm depth to the core surface (Figure 7c). The sediment δ^13^C profile changed little throughout the record (Figure 7c).

## Discussion

### Archeological interpretations – the legacy of Kukulek village on freshwater ponds

The sediment record from Kukulek Pond (nearest to the archeological site) showed isotopic and diatom assemblage shifts consistent with the pond experiencing nutrient inputs from the historical village (Figure 4a and c). The high δ^15^N values suggest that the elevated nutrients in the pond are due to marine-derived and/or trophically-enriched nutrients from the processing of marine mammals at the historical village. This interpretation is supported by archeological evidence documenting an abundance of marine mammal remains scattered throughout the site, including the frames of most dwellings that were constructed in part using whale or walrus bones (Geist and Rainey, 1936). Arctic lake sediments not receiving any marine input typically have δ^15^N values less than 5‰, which is notably lower than values recorded in the tissues of marine mammals (e.g. δ^15^N_bowhead whale_ = 13.3‰; δ^15^N_grey whale_ = 12.0‰; δ^15^N_walrus_ = 13.5‰; δ^15^N_spotted seal_ = 17.8‰; Dehn et al., 2006, 2007). In addition, many of the meat cache structures still contained meat, blubber, hides, and skin ropes at the time of excavation, and the soil around these structures was impregnated with oil from blubber to the point where the ground could be lit with a match (Geist and Rainey, 1936).

The δ^15^N values in the Kukulek sediment core have always been high relative to typical Arctic lake sediments, but they reach peak values following 5.25 cm depth (Figure 4c). Extrapolating the CRS model of ^210^Pb dates to this depth provides an age estimate of ca.1787 CE with a potential age range from 1553 CE to 1881 CE. We acknowledge that the ^210^Pb profile for this site should be viewed with caution due to low activities, and especially for older and extrapolated dates. Peak δ^15^N values may be expected with an increase in population and greater hunting activity resulting in more nutrients and their run-off from the site. However, there are no historical records from this period to corroborate a human population spike in the late 18th century, although the site is thought to have been near its maximum at the time of the famine (Mudar and Speaker, 2003). Archeological data present the possibility that the site was abandoned and resettled at this time as two houses were built during the Recent-Prehistoric phase at the Kukulek site (bracketed to have been built between 1649 and 1879 CE) and sod lines suggest a period of abandonment (Geist and Rainey, 1936). Alternatively, this sediment interval might be younger than the age-depth model suggests and may rather correspond to the late 19th century warming, evident in all the other pond records at ca. 1880 CE (discussed below; Figures 5–7). The interpretation of a warming signature at Kukulek is supported by slight increases in *Cladotanytarsus mancus*-type and *Psectrocladius* spp. (Figure 4b), which are considered to be thermally tolerant but often more abundant in shallow, warm lakes (Barley et al., 2006; Fallu et al., 2005; Heiri et al., 2011).

Despite a major reduction in hunting success during the famine, several factors may have caused an increase in runoff elevated in δ^15^N from the site. The scarcity of food would have resulted in extensive unearthing and raiding of multiple generations of meat caches. Additionally, houses that were abandoned were stripped for their hides and bone structures in a desperate bid for food (Krupnik and Krutak, 2002). Such a disturbance would expose these structures to increased weathering and thaw, hastening the release of marine-derived nutrients into Kukulek Pond. Finally, the grim reality of the famine included victims who succumbed to hunger and were found buried and unburied across the site (Geist and Rainey, 1936), which would have resulted in additional runoff elevated in δ^15^N values.

Although we view the extrapolated dates with caution, there are plausible explanations that indicate the peak δ^15^N values corresponds with the 1878–1880 CE famine. The sediment record has the highest δ^15^N values between 5.25 cm (inferred as the ~1880 CE famine) and 3.25 cm (~1930 CE), the latter of which approximately coincides with the excavation in 1934–35 CE. The archeological excavation would similarly expose previously buried, nutrient-rich soils and mammal remains. Additionally, disturbed soils and dug channels may have hastened surface water runoff during the brief growing season into nearby aquatic environments. The removal of some of the site material may also have driven the lowering of sedimentary δ^15^N values after ca. 1935 CE back toward pre-disturbance values (Figure 4c).

The Kukulek Pond diatom assemblages indicate elevated nutrients and more acidic conditions from 5.25 cm to the top of the core, a trend that becomes more pronounced above 3.25 cm depth (ca. 1930 CE; Figure 4a). The two significant zones at 5.25 and 3.25 cm (ca. 1930 CE) are coincident with the increases in δ^15^N values. The most notable taxonomic changes include an increase in *Nitzschia* genera, which, although common in Arctic ponds, tend to dominate in waters with elevated nutrients (Keatley et al., 2009) and often thrive in response to nutrient influxes like sewage runoff (Michelutti et al., 2002, 2007). Additionally, *Fistulifera* spp., an indicator of sewage inputs (Michelutti et al., 2007; Stewart et al., 2014), becomes more common (albeit at low abundances) coincident with elevated δ^15^N values. More acidic conditions are inferred with the increase in the acidophilic, benthic fragilarioid *Stauroforma exiguiformis* (Duda et al., 2020; Flower et al., 1996). The increase in *S. exiguiformis* may be reflecting greater allochthonous inputs, with the increased organic acidity giving rise to more acidic conditions in this poorly-buffered site (Kahl et al., 1989) and higher dissolved organic carbon (DOC) resulting in lower light penetration, giving *S. exiguiformis* a competitive advantage over other taxa.

### Elevated δ^15^N values at Reindeer pond

The elevated δ^15^N values (~11‰) at the base of the Reindeer Pond record (Figure 7c) were unexpected in a “reference site.” Sediment from the other reference site (Ollie Pond) had mean δ^15^N values (2.03 ± 0.3‰) consistent with runoff from relatively unenriched sources such as soils (mean δ^15^N values for Alaskan tundra soils 1.2 ± 0.3‰; Hobbie and Hobbie, 2006; δ^15^N 1.0 to 2.4‰; Xu et al., 2018), organic matter from permafrost thaw (δ^15^N 1.0 ± 0.1‰ Xu et al. (2018), and plant matter (δ^15^N 0 to −11‰ Hobbie et al., 2000) and the principal vector of autochthonous nitrogen is through nitrogen fixation by cyanobacteria (with a fractionation factor of −0.55‰ Minagawa and Wada, 1984). Although microbial activity can enrich organic matter, the extreme cold environment and saturated conditions of most tundra soils lead to slower decomposition and, consequently, lower δ^15^N values as compared to temperate or tropical soils (Xu et al., 2018). Additionally, pond sediments likely integrate many nitrogen pools so that, while fractionation may occur (e.g. between ammonium and nitrate (Talbot, 2001), or between mycorrhizal fungi and associated plants (Hobbie et al., 2000)), the δ^15^N values of the sediment should largely reflect the isotopic composition of the original source pool.

Three potential explanations for the enriched signature in Reindeer Pond prior to the late 19th century are: (1) a previously unknown settlement; (2) a pre-historic bird colony; and (3) denitrification. However, each of these potential explanations are problematic. Reindeer Pond is unlikely to have had a hunting camp or settlement during the pre-1880 CE period as it is far from the coast and the only reliable inland source of meat is reindeer, which were only introduced in 1900 CE (after the elevated δ^15^N values) and, compared to marine mammals, have relatively low δ^15^N values (e.g. mean δ^15^N_deer_ = 5.3‰ vs δ^15^N_walrus_ = 13.5‰; Dehn et al., 2007; Price and Burton, 2011). Marine-derived nutrients could also enter the pond from a bird colony within the catchment (e.g. Duda et al., 2020; Stewart et al., 2013). The diatom record shows a slight increase in *Nitzschia perminuta*, a taxon that often dominates high nutrient sites in the Arctic (Keatley et al., 2009), in this earliest part of the record (Figure 7a). However, the low values of chl *a*, %N and %C (Figure 7c) are not consistent with high nutrient inputs. Finally, denitrification, which has a high enrichment factor of (+5‰ to +40‰ Kendall (1998); +20‰ Talbot (2001), by anaerobic bacteria living in sustained low oxygen conditions under extensive ice, might also explain the high values. However, anaerobic conditions are unlikely as Reindeer Pond is relatively shallow, such that open water should develop fairly rapidly with ice off, and extensive light and wind mixing during the open water season should keep benthic waters oxygenated (Stewart et al., 2013). In addition, the pre-1880 CE chironomid assemblage (Figure 7b) does not suggest low oxygen conditions (Brodersen et al., 2004; Cranston, 1995). Consequently, the pre-1880 elevated δ^15^N values values remains enigmatic. The decline in the δ^15^N values for Reindeer Pond after 1880 CE, coincident with an increase in primary production (Figure 7c), may reflect the input of sources lower in δ^15^N values coming from the catchment or the proliferation of nitrogen-fixing organisms in response to a longer ice-free season.

### The influence of climate change on freshwater ponds at St. Lawrence Island

Reindeer Pond (Highland reference site) – The paleoenvironmental records from the reference ponds on St. Lawrence Island suggest that the region has been experiencing warming since ca. 1880 CE. In Reindeer Pond, a marked increase in sediment chl *a* (Figure 7c) beginning ca. 1880 CE suggests longer growing seasons associated with rising temperatures as documented by instrumental records of air temperature since 1927 CE when monitoring began (Figure 2). A coincident shift in chironomid assemblages at ca. 1880 CE (Figure 7b) is marked by the disappearance or decline of several cold-stenotherms (*Arctodiamesa* spp., *Thienemanniella* -type E, and *Mesocricotopus* spp.) and increases in warmer, littoral taxa (*Cricotopus*-type P, *Cricotopus intersectus*-type, *Metriocnemus* spp., *Psectrocladius sordidellus*-type, and Tanytarsini spp.) including macrophyte-associated species (*Cricotopus*-type P, *Cricotopus intersectus*-type; Brodersen et al., 2001; Cranston et al., 1983) suggesting the establishment of a complex, vegetated littoral zone. Chironomid life cycles are dependent on temperature and, as such, assemblages often respond directly to changes in water temperature (Eggermont and Heiri, 2012).

The diatom assemblages from Reindeer Pond show moderate increases in the eurytopic *Achnanthidium minutissimum* (Lim et al., 2001; Van Dam, 1996), with relative decreases in the acidophilic *Stauroforma exiguiformis* (Flower et al., 1996) and *Fragilaria capucina* (Figure 7a); a taxonomic shift that has been recorded elsewhere in Arctic ponds experiencing warming (Antoniades et al., 2005; Douglas et al., 1994). Reindeer Pond is located at a higher elevation than the lowland sites, is farther from the mediating effect of the coast, and has a rockier catchment compared to all other study ponds. If past ice cover was longer in duration and more restrictive to growth than at present, the diatom assemblages in Reindeer Pond would be expected to show a marked response, particularly with respect to new habitats (e.g. more aquatic vegetation) that occur with warming (Griffiths et al., 2017). However, diatom changes in Reindeer Pond are muted, although similar in nature, compared to changes recorded in High Arctic sites (e.g. Antoniades et al., 2005; Douglas et al., 1994) and the pre-warming assemblage is also more diverse, suggesting that the pre-1880 CE ice-free season was likely long enough to allow for relatively higher diversity of diatom habitats. With warming, however, the record suggests that there has been an increase in habitat complexity, or expansion into novel habitats, and increased nutrients, possibly from increased thawing of permafrost (Reyes and Lougheed, 2015), or increased vegetation establishment in the catchment.

Ollie Pond (Lowland reference site) – The lowland, vegetated reference Ollie Pond has also experienced biotic shifts likely in response to changes in climate (Figure 6). Beginning in the 1880s, with the principal shift occurring in the 1920s, *Zalutschia*-type B declines in relative abundance, with corresponding increases in the low oxygen-tolerant *Chironomus anthracinus* and *C. plumosus* (Brodersen et al., 2004; Hamburger et al., 1994; Walshe, 1950), and macrophyte-associated *Corynoneura* spp. (Cao et al., 2014; Figure 6b). The assemblage shift suggests a transition to warmer conditions, as *Chironomus* spp. have higher temperature optima than *Zalutschia-*type B (Barley et al., 2006; Bennike et al., 2004). As noted earlier, what we identified as *Zalutschia-*type B, a European morphotype, is more likely *Zalutschia* nr *lingulata pauca*, which is also a cold water taxon (Medeiros et al., 2022). Additionally, the increase in *Chironomus* spp. may be reflecting reduced oxygen levels in the benthos, as these species are oxy-regulators and consequently have a relatively high tolerance to low oxygen conditions (Brodersen et al., 2004; Brodersen and Quinlan, 2006). Arctic ponds are generally well-oxygenated in the short Arctic summers (e.g. Stewart et al., 2013); however, colored dystrophic systems like Ollie Pond (DOC = 10.6 mg/L) can stratify for days, or even the whole season (Breton et al., 2009), due to their greater absorption of light energy, leading to periodic hypoxia or even localized anoxia. Low oxygen conditions in Ollie Pond may be compounded by greater permafrost thaw in the catchment, which can lead to increased DOC and nutrient loading into the tundra ponds (Breton et al., 2009; Lougheed et al., 2011; Rautio et al., 2011; Reyes and Lougheed, 2015).

Ollie Pond recorded a muted, although predictable diatom assemblage response to recent climate warming (Figure 6a). Slight increases in the relative abundances of the eutraphentic *Eolimna minima* (Moravcová et al., 2013), the epipsammic *Psammothidium rossii*, and eurytopic *Achnanthidium minutissimum* (Lim et al., 2001; Van Dam, 1996) around 1950 CE, and the increase and *Diatoma tenuis*, around 1980 CE, suggest increasing nutrients and conductivity in Ollie Pond. *Diatoma tenuis* is found in higher conductivity waters (Ryves et al., 2002) and may be responding to either increased evaporation, concentrating solutes in the pond (a trend occurring throughout the circumpolar Arctic; Riordan et al., 2006; Smol and Douglas, 2007), or increased storm activity and sea spray from the Bering Sea. The limnology of Ollie Pond (Table 1) is similar to the other coastal ponds on St. Lawrence Island, suggesting the increase in *D. tenuis* is unlikely to be from anthropogenic activities in the town of Savoonga. The Ollie Pond sedimentary chl *a* profile (Figure 6c) suggests that primary production is low, but has been slightly increasing at the lowland site through the entire record and is accompanied by corresponding increases in %C and %N. The high DOC in deep Ollie Pond may be precluding a marked rise in primary production with warming (as inferred at the other sites), as the limiting factor on production may be light penetration (Karlsson et al., 2009). The potentially elevated DOC with permafrost thaw (Reyes and Lougheed, 2015) and resulting reduced light penetration to the deeper areas of Ollie Pond, may be reducing the available habitat for diatom and macrophyte growth and inhibiting benthic production relative to a similar-sized, clear-water pond, like Reindeer Pond. The isotopic records of δ^15^N and δ^13^C values show little directional change through the profile (Figure 6c), suggesting consistent sources of carbon and nitrogen to Ollie Pond.

Green Pond (Potentially impacted site) – Green Pond and the impacted Kukulek Pond were thought to be hydrologically-connected during periods of elevated water levels; however, the sedimentary profiles suggest different trajectories for the two sites and hint at little-to-no-connectivity between the waterbodies. The lack of connection is evident in the different δ^15^N records, with elevated δ^15^N values (average δ^15^N: 12‰) in Kukulek Pond (Figure 4c), reflective of marine-derived nutrient inputs, versus much lower values in Green Pond (average δ^15^N: 1.8‰; Figure 5c). The principal change in the chironomid record at Green Pond is an increase in the relative abundance of the littoral *Tanytarsus* spp. and a slight decrease in *Zalutschia* spp. (Figure 5b), which is associated with dystrophic waters (Saether, 1979), and is often the dominant taxon in coastal peat pools and shallow Arctic lakes (Barley et al., 2006; Walker et al., 1985). The Green Pond diatom record indicates a fairly substantial change in the dominant taxa in the late 19th century, when many aerophilic (*Diadesmis* spp., *Chamaepinnularia soehrensis*; Johansen, 2010; Wojtal, 2009) and macrophyte-associated taxa (*Pinnularia* spp.; Douglas and Smol, 1995; Lim et al., 2001) were supplanted by epipsammic (*Psammothidium* spp.) and acidophilic (*Eunotia* spp.; DeNicola, 2000) taxa (Figure 5a). This taxonomic shift is accompanied by an increase in primary production and a transition from variable carbon and nitrogen concentrations to more stable values (Figure 5c). Unlike the clear nutrient signal identified in Kukulek Pond, the Green Pond record suggests a transition from a potentially rich fen-like environment to a tundra thaw pond.

Two potential mechanisms behind the above diatom transitions could be a raising and stabilization of the water table due to higher precipitation or due to ground subsidence, and the formation of a thaw pond in response to warming temperatures. For the western Alaska region, winter, spring, and fall precipitation has been generally increasing, although with high variability, over the past 50 years (from 1949 to 1998), while summer precipitation has been decreasing (Figure 2; McAfee et al., 2014; Stafford et al., 2000). In areas of discontinuous permafrost (like St. Lawrence Island) there has been an increase in the formation of thermokarst features as a result of permafrost thaw (Yoshikawa and Hinzman, 2003) and the steep-sided morphology of Green Pond suggests that it may have formed as a result of permafrost degradation (Jorgenson and Shur, 2007). Warming-induced permafrost thaw has resulted in 771 of St. Lawrence Island’s 3271 water bodies experiencing drainage events, which is ⁓40 times higher than the rate recorded across the entire northern permafrost region (Liu et al., 2024). The rapid transition from dominantly aerophilic to littoral diatom taxa similarly indicates rapid pond expansion. The timing of this transition suggests that the permafrost degradation may have been caused by the early 19th century warming on St. Lawrence Island.

Kukulek Pond (Impacted site) – In Kukulek Pond, the isotopic and diatom species changes at 5.25 cm are coeval with the subtle shifts in the chironomid assemblages, including a relative increase in the warm stenothermic *Cladotanytarsus mancus*-type (Barley et al., 2006; Bennike et al., 2004) and the decline in the cold stenotherms *Hydrobaenus/Oliveridia* spp. (Barley et al., 2006; Bennike et al., 2004). The comparatively shallow nature of Kukulek Pond (⁓1/3 the depth of Ollie Pond) would make stratification unlikely, despite the high DOC. Additionally, light, although attenuated, would likely reach the benthos leading to oxygenated bottom waters in the summer, and the general decline of *Chironomus* spp. in the uppermost sediments may rather be due to increases in other taxa, such as the littoral, acidophilic *Psectrocladius* spp. The timing of these paleoenvironmental changes at Kukulek Pond are roughly coincident with the 1880 CE famine and abandonment of the village.

The diatom-inferred increases in nutrients and reduction of pH in Kukulek Pond are coincident with the two periods of site disturbance at the Kukulek site and with the chironomid-inferred late 19th century warming on St. Lawrence Island, making the disentangling of these two events difficult. For example, after 1935 CE, sedimentary δ^15^N values decline in Kukulek Pond, which may, in part, be a response to the removal of the many artifacts and bone structures as a result of the excavation. However, the lack of corresponding declines in primary production or in the relative abundances of nutrient-associated taxa suggest that other factors may also have played a role. Nitrogen fixation by benthic cyanobacteria, which are common in Arctic lakes (Vincent, 2000), has a slightly negative associated fractionation (−0.55‰; Minagawa and Wada, 1984). The water chemistry suggests that Kukulek Pond was nitrogen-limited at the time of sampling (Table 1) and the proliferation of nitrogen-fixing cyanobacteria taking advantage of the excess proportion of phosphorus in the system and the lengthened growing season due to warming may be contributing to the slight decline in δ^15^N values.

The Kukulek Pond diatom assemblage shifts may be reflecting an interaction between climate and nutrient inputs from the archeological site. For example, the nutrient-associated taxa increase in concert with the δ^15^N changes. However, the increases in more eutrophic diatom taxa are continuous and do not decline after the 1930s excavation, suggesting that warming may be partially mediating nutrient release at Kukulek, likely through increased permafrost thaw (Hobbie et al., 1999; Liu et al., 2024). The increase in whole lake production is also coincident with the warming signal; however, the signal is not as marked as in the upland reference site (Reindeer Pond). Primary production at Kukulek Pond is likely responding to a combination of increased nutrients from disturbance and permafrost degradation, the lengthening of the growing season, and may have been partially tempered by increased DOC limiting light availability (Karlsson et al., 2009).

The impacts on aquatic ecology from the archeological site adjacent to Kukulek Pond cannot be interpreted in isolation of 20th century changes in climate. The mobilization of nutrients and DOC from the site with permafrost thaw are likely sustaining the relatively higher primary production and nutrient-associated diatom taxa, decades after the last significant period of site disturbance. This suggests that Arctic ponds in regions such as this, where labile nutrients previously entombed in permafrost are now being released, may be the most prone to ecological change with continued warming.

## Conclusions

This study is one of a growing number that strategically combines paleolimnology with archeological investigations. The impact of Kukulek village was evident in the elevated δ^15^N values and diatom shifts recorded in the nearby Kukulek Pond core. Although our record was of relatively short duration, the biota may have responded to the occupation of the site and this biotic signal was used to identify periods of disturbance, particularly the inferred 1878–1880 CE famine and the 1934–1935 CE archeological excavations. Additionally, the sediment core data provided missing environmental context for the abandonment of the Kukulek village. In particular, the coincident timing of the famine with the onset of warming conditions on St. Lawrence Island merits further investigation.

The paleolimnological records from St. Lawrence Island also record changes in aquatic biota consistent with warming around 1880 CE. The biotic signal generally reflects increased primary production, the opening of new habitats for colonization, and shifts toward chironomid taxa adapted to warmer waters. However, these changes are moderated by the local limnological conditions where factors such as light penetration, nutrient influx, and physical changes to the pond morphometry due to permafrost thaw, induced variability in the biotic response. This suggests that lakes and ponds located in the permafrost may be subject to possible synergistic impacts of warming and nutrient loading due to anthropogenic climate change.

The diatom and chl *a* profiles from Ollie and Kukulek ponds, as well as the possible thermokarst formation of Green Pond, suggest that the coastal sites are being impacted by permafrost thaw. This has implications for the two permanent coastal communities on St. Lawrence Island and the many semi-permanent camps, as permafrost degradation could impact infrastructure as well as potentially release nutrients and contaminants into the surface water and possibly groundwater. With a warming climate, the impacts to northern communities are going to be more acute, particularly given their reliance on natural and marine resources for subsistence and income.

## Supplemental Material

sj-xlsx-1-hol-10.1177_09596836261477277 – Supplemental material for Using lake sediments to reconstruct the indigenous occupation of the Kukulek archeological site and the impact of recent climate change on freshwater ecosystems at St. Lawrence Island, Alaska Supplemental material, sj-xlsx-1-hol-10.1177_09596836261477277 for Using lake sediments to reconstruct the indigenous occupation of the Kukulek archeological site and the impact of recent climate change on freshwater ecosystems at St. Lawrence Island, Alaska by Neal Michelutti, Katherine Griffiths, Jules M. Blais, Linda E. Kimpe, Janae Rampone, Joshua Kurek and John P. Smol in The Holocene

sj-xlsx-2-hol-10.1177_09596836261477277 – Supplemental material for Using lake sediments to reconstruct the indigenous occupation of the Kukulek archeological site and the impact of recent climate change on freshwater ecosystems at St. Lawrence Island, Alaska Supplemental material, sj-xlsx-2-hol-10.1177_09596836261477277 for Using lake sediments to reconstruct the indigenous occupation of the Kukulek archeological site and the impact of recent climate change on freshwater ecosystems at St. Lawrence Island, Alaska by Neal Michelutti, Katherine Griffiths, Jules M. Blais, Linda E. Kimpe, Janae Rampone, Joshua Kurek and John P. Smol in The Holocene

## Data Availability

All data are available in Supplemental Materials.
